# Reddish multilobulated mass on the scalp in a 13‐year‐old girl

**DOI:** 10.1002/ccr3.5189

**Published:** 2021-12-26

**Authors:** Mohamed Ben Rejeb, Randa Said El Mabrouk, Sirine Ben Cheikh, Badreddine Sriha, Nejet Ghariani, Mohamed Denguezli

**Affiliations:** ^1^ Dermatology Department Hached University Hospital of Sousse Sousse Tunisia; ^2^ Anatomopathology Department Hached University Hospital of Sousse Sousse Tunisia

**Keywords:** adnexal tumors, nodular hidradenoma, pediatric dermatology, sclap

## Abstract

Nodular hidradenoma is a rare benign adnexal tumor that occurs as a solitary nodule with a predilection for the head, face, and upper extremities. We describe the case of nodular hidradenoma presenting as an expanding nodule on the scalp. This tumor is a simulator of malignant lesions on clinical and dermoscopic examination. Our case emphasizes the importance to identify this tumor as a differential for scalp lesions.

## Graphical abstract

Clinicians should be aware of NH as a possible differential diagnosis of cutaneous tumors of the scalp affecting both children and adults. As there are no specific criteria about NH, the final diagnosis should take into account the clinical, dermoscopic, and histopathological findings.

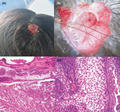

## 1 CASE PRESENTATION

**FIGURE 1:**
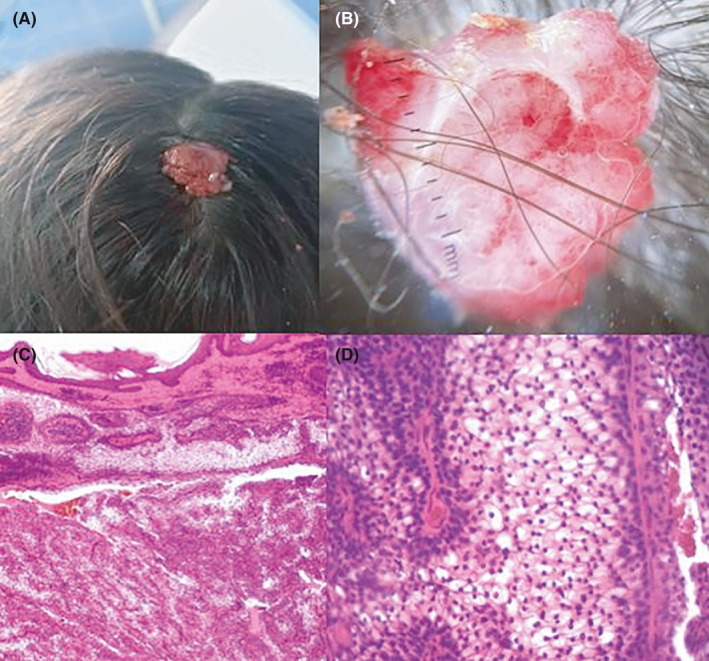
A, Reddish multilobulated fleshy mobile mass over the vertex of the scalp (4.0 × 3.0 cm), B a dermoscopic pattern composed of a reddish‐white homogeneous area with white shiny lines and numerous glomerular and dotted vessels: a dermal well‐circumscribed, unencapsulated epithelial tumor composed of cystic spaces filled with eosinophilic material (C, HE_*_40) and a proliferating cells with clear cytoplasm without mitotic images (D, HE_*_200)

A 13‐year‐old female patient presented with a 2‐year history of solitary, painless, slow‐growing, sessile, mobile, multilobulated mass 4.0 × 3.0 cm in size located above the vertex of the scalp (Figure 1A). Dermoscopic examination showed a pattern composed of a white‐reddish homogeneous area, with crysalids, red lacuna, and numerous glomerular and dotted vessels (Figure 1B). Histopathological examination revealed dermal well‐circumscribed, unencapsulated epithelial tumor composed of proliferating cells with clear cytoplasm and cystic spaces filled with eosinophilic material without mitotic images (Figure 1C,D). These findings are consistent with a diagnosis of nodular hidradenoma (NH).

## 2 DISCUSSION

Nodular hidradenoma is an uncommon benign epithelial neoplasm arising from eccrine sweat gland that usually appears in adults and extremely rare in children. NH lacks any distinctive clinical features but is usually present as a solitary, well‐circumscribed, slowly growing, nodular lesion most commonly located over the head and trunk.^1^ Due to its clinical heterogeneity, dermoscopy could make easier the diagnosis of NH. The most common dermoscopic pattern is composed of a homogeneous pink area, white shiny lines, and vascular structures with linear, dotted, and hairpin vessels.^2^ Because NH is a clinical simulator of other tumors, this case underscores the importance of a clinical, dermoscopic, and anatomopathological correlation for a diagnosis.

## CONFLICT OF INTEREST

None.

## AUTHOR CONTRIBUTIONS

Ben Rejeb Mohamed involved in writing the manuscript and submitting the revised article. Randa Said El Mabrouk collected the images and analyzed the dermoscopic part of manuscript. Ghariani nejet carried out the analysis and corrected the manuscript. Ben chikh sirine involved in writing the histological part of the manuscript. Sriha Baderddine reviewed the histological part of manuscript. Denguezli mohamed supervised and approved the revised manuscript.

## ETHICAL APPROVAL

Ethics statement for this article was approved.

## CONSENT

The consent statement was approved by all the authors. Written informed consent was also obtained from the patient and her family to publish this report in accordance with the journal's patient consent policy.

## Data Availability

Data are openly available in a public repository that issues datasets with DOIs.
